# Morphological specificity analysis of an image-based 3D model of airway filling in a difficult airway

**DOI:** 10.1186/s12871-022-01880-6

**Published:** 2022-11-03

**Authors:** Wendong Chen, Li Ma, Jianlin Shao, Chun Bi, Yuchen Xie, Shuangyan Zhao

**Affiliations:** Department of Anesthesiology, The First Affiliated Hospital of Kunming Medical University, No 295 Xichang Road, Wuhua District, Kunming City, Yunnan Province 650032 People’s Republic of China

**Keywords:** Difficult airway, Three-dimensional model, Upper airway, Morphological

## Abstract

**Background:**

The purpose of this study was to analyze position-specific morphological changes of the upper airway and to further assess the impact of these changes in difficult airway during intubation.

**Methods:**

This observational comparative study included two groups (*n* = 20 patients/group): Group A had normal airway and Group B had difficult airway. Data obtained from two-dimensional magnetic resonance imaging were imported to Mimics V20.0 software for processing. We then reconstructed three-dimensional models of upper airway filling in patients in the supine and maximum extension position based on the imaging data. Those models were projected on coronal, sagittal, and horizontal planes to investigate multiple morphological features. We measured the surface area, radial length, and corner angle of the projected areas.

**Results:**

Group A had larger upper airway filling volumes compared to Group B The volumes for the supine position were 6,323.83 ± 156.06 mm^3^ for Group A and 5,336.22 ± 316.13 mm^3^ for Group B (*p* = 0.003). The volumes the maximum extension position were 9,186.58 ± 512.61 mm^3^ for Group A and 6,735.46 ± 794.63 mm^3^ for Group B (*p* = 0.003). Airway volume increased in the upper airway filling model as the body position varied from the supine to maximum extension position (Group A: volume increase 2,953.75 ± 524.6 mm^3^, rate of change 31%; Group B: volume increase 1,632.89 ± 662.66 mm^3^, rate of change 25%; *p* = 0.052).

**Conclusion:**

The three-dimensional reconstruction model developed in this study was used to digitally quantify morphological features of a difficult airway and could be used as a novel airway management assessment tool.

**Supplementary Information:**

The online version contains supplementary material available at 10.1186/s12871-022-01880-6.

## Background

The clinical definition of a difficult airway varies widely in the literature [1]. Previous practice guidelines described a difficult airway as one which a professionally trained anesthesiologist with more than five years of experience in clinical anesthesia encounters during upper airway mask ventilation, tracheal intubation, or both [1]. However, more recent guidelines suggest that difficult airway include difficulties with the following: mask ventilation, ventilation on the glottis, placing the tool on the glottis, exposing the laryngoscope, intubation of the trachea, and failure to repeat intubation [2]. Globally, approximately 70% of anesthesia-related mortality is due to difficult airway [3]. Potential difficulties with intubation during anesthesia are often unpredictable or challenging to estimate [4, 5]. The occurrence of a difficult airway cannot be completely ruled out during the induction of anesthesia even after a technical assessment has been performed [6]. Moreover, a patient would be at high risk if an unexpected difficult airway presents after induction of general anesthesia [7].

Clinical assessment techniques developed by Mallampati et al., and Cormack–Lehane et al., have been in use for more than 20 years [8, 9]. Some published studies reported weaknesses in Mallampati’s techniques for evaluating a difficult airway [10], and the Wilson scoring method has a reported sensitivity of 75% [11]. Although oropharyngeal volume is associated with difficult intubation [12], the Mallampati score itself is still not sufficient to predict difficulty during tracheal intubation [10]. These studies suggest that the usual prediction methods for difficult airway lack reliability and accuracy [13].

The purpose of this observational study was to develop a digital technique for assessing morphological changes in difficult airway when a patient moves from a supine to a maximum extension position during intubation. We tested the hypothesis that a digital technique could better identify morphological changes in difficult airway, providing more accurate and reliably quantifiable evidence for evaluating potential difficulties during airway intubation.

## Materials and methods

The protocol was reviewed and approved by the ethics committee of the First Affiliated Hospital of Kunming Medical University. Informed signed consent was provided by all study participants, including study participation and the publication of identifying images in an online open-access publication. All methods were carried out in accordance with relevant guidelines and regulations.

### Research subjects

Inclusion criteria were as follows: 1) informed consent was obtained from the volunteers and their family members; 2) adults with no history of maxillofacial surgery or trauma who were able to cooperate during magnetic resonance imaging (MRI) examination; and 3) no MRI relative and absolute contraindications. Exclusion criteria were as follows: 1) patients or their family members requested withdrawal during the study; and 2) simultaneous participation in other clinical interventions.

**Fig. 1:**
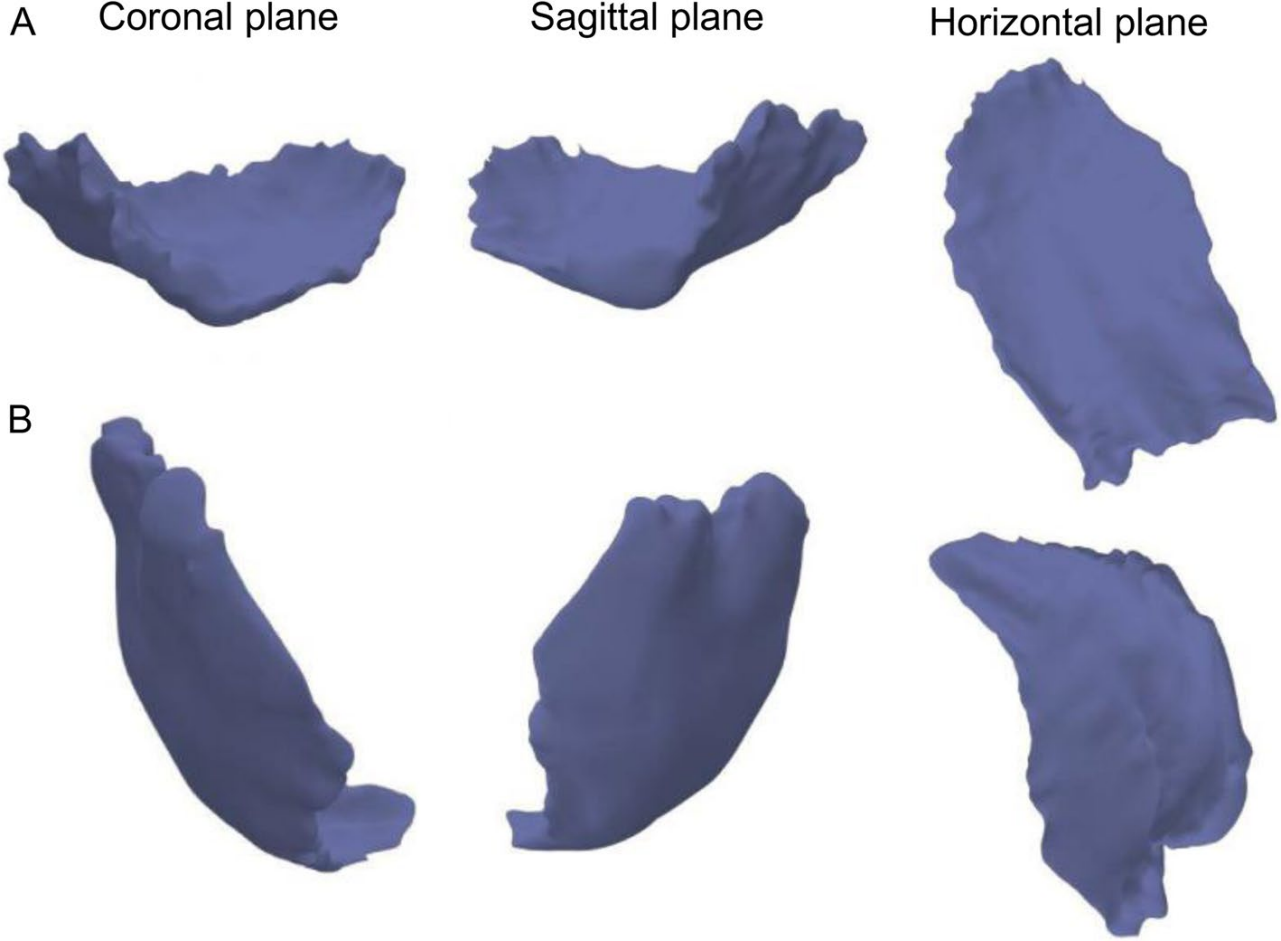
3D model of filling of the upper airway in (**A**) the supine position and (**B**) the maximum extension position

We applied MimicsV20.0 software to reconstruct the upper airway filling of a three dimensional (3D) finite element model based on two dimensional (2D) MRI images. The validity of the 3D finite element model of the upper airway was verified. Before exporting, 2D image data were imported to Mimics V20.0 software, and then a geometric mask was edited and imported using reverse engineering software for polishing and smoothing. The normal airway group (Group A) and difficult airway group (Group B) each included 20 patient models. The criteria for normal airway were as follows: Mallampati grade I-II, mouth opening > 4 cm, thyromental distance (TMD) > 6 cm and normal head and neck mobility, endotracheal intubation was successfully performed once, and the total time from laryngoscope placement to confirming the position of endotracheal tube was < 30 s. A difficult airway was judged as: Mallampati grade III to IV, mouth opening < 4 cm, TMD < 6 cm, reduced range of motion of head and neck, intubation failed after three consecutive attempts with direct laryngoscopy or > 10 min, or normal oxygenation could not be maintained with mask oxygen. The 3D model of upper airway filling was reconstructed using MRI data using the commercially available Mimics software (Mimics V20.0, Materialise Belgium) (Fig. 1).

### Measurements of relevant morphological data in the 3D model

#### Upper airway 3D model volume data measurement

*V*_*1a*_ and *V*_*2a*_ (Table 1) represent the volume data for Group A in the supine and maximum extension positions (*V*_*1b*_ and *V*_*2b*_ represent Group B). The definition of the supine position is shown on the left side of Supplementary Fig. 1A and briefly described here: When the patient is supine, the three axes of the oral (OA), pharynx (PA), and larynx (LA) cross each other, and the path from the incisor teeth to the vocal portal is almost perpendicular to the axis of the larynx. The definition of the maximum extension position is shown in Supplementary Fig. 1B and briefly described here: When the patient is supine with the head in the "sniffing position" the head is tilted back at the atlanto-occipital joint, resulting in a near overlap of the three axes of the OA, PA, and LA. As such, the path from the incisor teeth to the vocal door is almost straight. A professional surveyor conducted the volume measurements in the normal and difficult airway based on the 3D models (Fig. 1). The specific process of measurement involved the 3D finite element model and volume measurement of upper airway filling in the supine position; the 3D finite element model and volume measurement of upper airway filling occurred in the maximum extension position.

**Table 1:** Nomenclature

*V*_*1a*_	*V*_*1a,*_: model volume at supine position (Group A);
*V*_*2a*_	*V*_*2a*_*,*: model volume at maximum extension position (Group A);
*V*_*1b*_	*V*_*1b,*_: model volume at supine position (Group B);
*V*_*2b*_	*V*_*2b*_*, *_*y*_: model volume at maximum extension position (Group B);
*L*_*SLH*_/*L*_*ELH*_	The longitudinal radial length of the model on the horizontal projection plane at the supine position/at the maximum extension position
*L*_*SLS*_/*L*_*ELS*_	The longitudinal radial length of the model on the sagittal projection plane at the supine position / at the maximum extension position
*L*_*SLC*_/*L*_*ELC*_	The longitudinal radial length of the model on the Coronal projection plane at the supine position/ at the maximum extension position
*L*_*STH*_/*L*_*ETH*_	The transverse radial length of the model on the horizontal projection plane at the supine position/ at the maximum extension position
*L*_*STS*_/*L*_*ETS*_	The transverse radial length of the model on the sagittal projection plane at the supine position/ at the maximum extension position
*L*_*STC*_/*L*_*ETC*_	The transverse radial length of the model on the coronal projection plane at the supine position/ at the maximum extension position
*S*_*SH*_/ *S*_*EH*_	The area of the model on the horizontal plane at the supine position/ at the maximum extension position
*S*_*SS*_/ *S*_*ES*_	The area of the model on the Sagittal plane at the supine position/ at the maximum extension position
*S*_*SC*_/ *S*_*EC*_	The area on the coronal plane of the model at the supine position/ at the maximum extension position
*α *_*SIH*_/ *α *_*EIH*_	The lower corner angle of the quadrilateral of the model on the horizontal projection plane at the supine position/ at the maximum extension position
*α *_*SIS*_/ *α *_*EIS*_	The lower corner angle of the quadrilateral of the model on the sagittal projection plane at the supine position/ at the maximum extension position
*α *_*SIC*_/ *α *_*EIC*_	The lower corner angle of the quadrilateral of the model on the coronal projection plane at the supine position/ at the maximum extension position
*α *_*SLH*_/ *α *_*ELH*_	The left corner angle of the quadrilateral of the model on the horizontal projection plane at the supine position/ at the maximum extension position
*α *_*SLS*_/ *α *_*ELS*_	The left corner angle of the quadrilateral of the model on the Sagittal projection plane at the supine position/ at the maximum extension position
*α *_*SLC*_/ *α *_*ELC*_	The left corner angle of the quadrilateral of the model on the ccoronal projection plane at the supine position/ at the maximum extension position

#### Upper airway 3D model projection data measurement

**Fig. 2:**
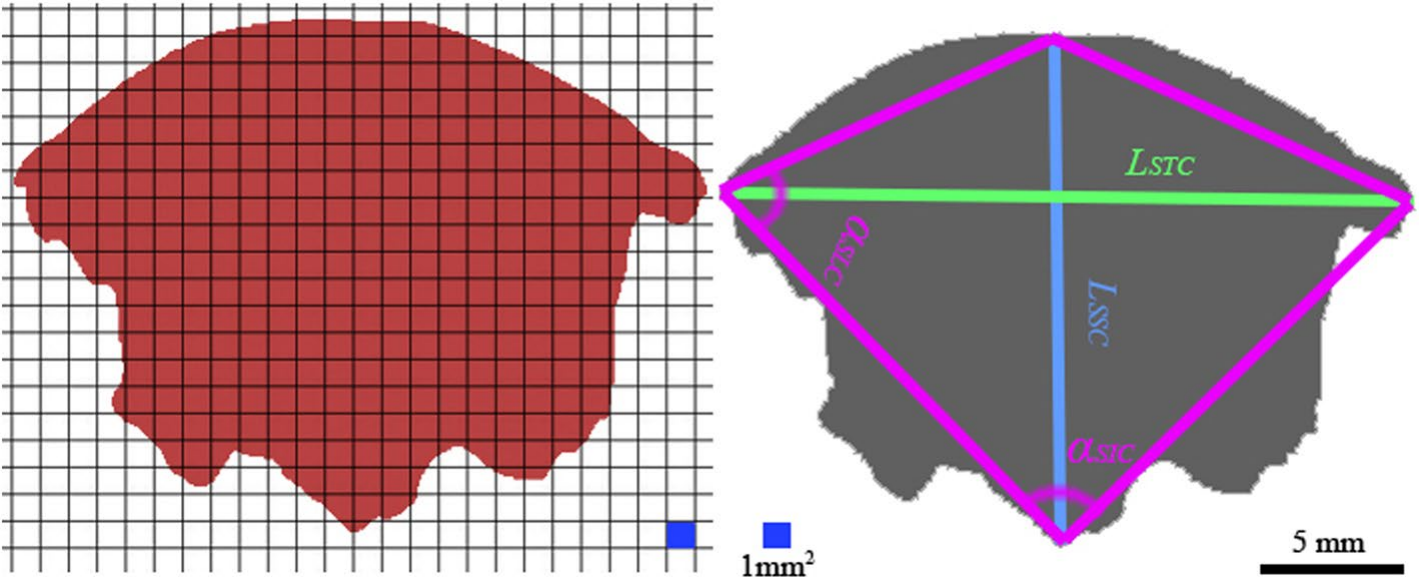
2D projection and data measurement of a 3D model of upper airway filling in the coronal plane

**Fig. 3:**
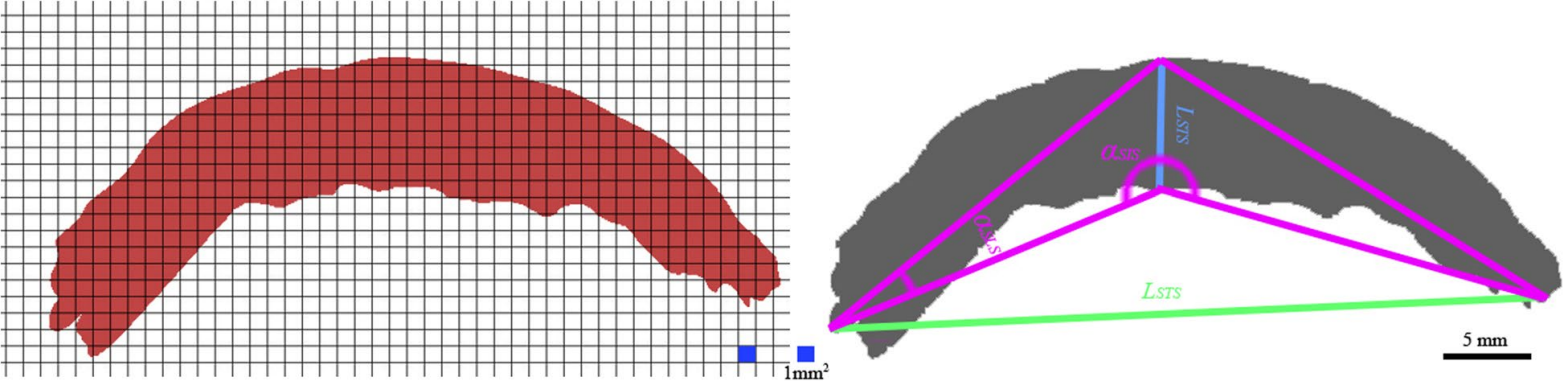
2D projection and data measurement of a 3D model of upper airway filling in the sagittal plane

**Fig. 4:**
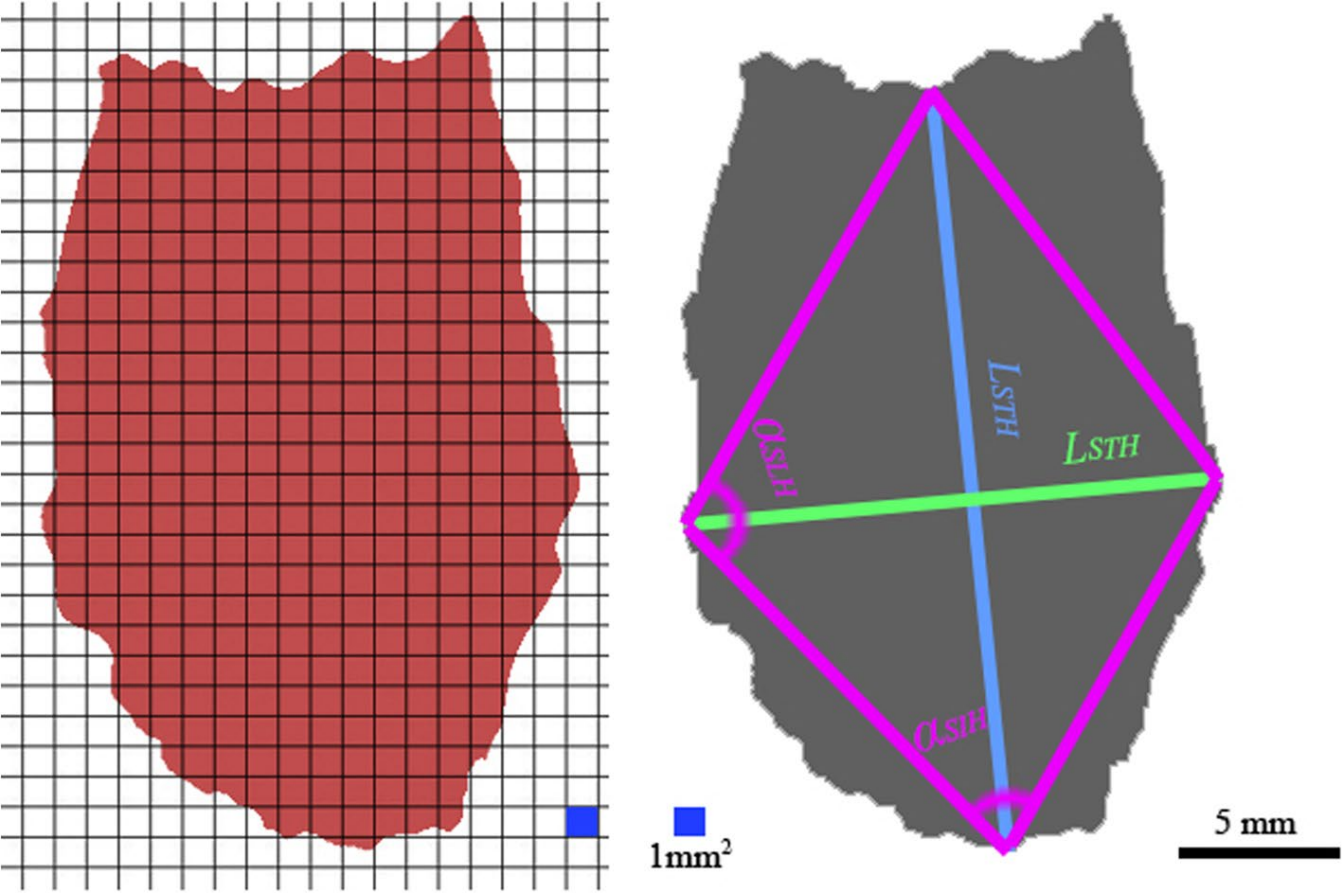
2D projection and data measurement of a 3D model of upper airway filling in the horizontal plane. Note: Volume in Group A is represented as *V*_*1a*_ and *V*_*2a*_; volume in Group B is represented as *V*_*1b*_ and *V*_*2b*_

We used 3-Matic (Materialise Belgium) to perform orthographic projections of the upper airway model to obtain geometric detail of the coronal, sagittal, and horizontal planes. The 2D projection surface was divided into numerous 1 mm^2^ square cells using Matlab (Mathworks, US). The mesh technique was used to improve measurement accuracy as previously reported [14]. Briefly, the mesh technique uses 2D and 3D geometry to calculate and analyze the geometric surface area, stress, displacement, etc. We marked four key points to represent four corner points based on meshing cells on each projected 2D image [15]. Two points along the transverse direction and another two points along the longitudinal direction were selected to measure maximum radial length in two directions. Those points generated a quadrilateral shape to simplify the surface shape for measurement purposes. The values of surface area and two corner angles of the quadrilateral were measured and included in the morphological dataset. Table 1 lists all measurement indexes and their definitions. We marked each measurement index on each projected 2D image (Figs. 2, 3 and 4). All measurements were carried out by one researcher and repeated three times.

### Statistical analysis

All measurement data are represented as mean ± standard deviation, and data were analyzed using MS Excel 2019 (Microsoft Corporation) and SPSS 19.0 (IBM Corporation) statistical software. The measurement data were analyzed using the two-tailed *t*-test. We performed a power analysis of hypothesis testing at the 0.05 level. In the two-tailed case when the sample size was 20 cases, we set the effect sizes as 0.2, 0.5, and 0.8, corresponding to the statistical power of 0.09, 0.33, and 0.69. These indicated that the larger the effect size, the higher the statistical power. Between-group differences were considered statistically significant at *p* < 0.05.

## Results

### Patient information

A total of 40 patients were selected and included in this study: 20 patients with normal airway (Group A) and 20 patients with difficult airway (Group B). Group A characteristics were as follows: male/female (14/6), age (39 ± 3.6) years, ASA class II/III (18/2), and body mass index (BMI) (21.3 ± 2.5) kg/m^2^. Group B characteristics were as follows: male/female (17/3), age (41 ± 2.9) years, ASA class II/III (19/1), and BMI (23.8 ± 6.2) kg/m^2^.

### Volumetric variation from the upper airway filling 3D model

Airway volume was significantly different between Group A and Group B (Table 2). Patients in Group A had greater upper airway volume compared to patients in Group B (*p* = 0.003) when moving from the supine position to the maximum extension position.

**Table 2:** Volume of an upper airway filling model in the supine and maximum extension positions

Item	Group A (mm^3^)	Group B (mm^3^)	*p*-value
Supine position (*V*_*1a*_, *V*_*2a*_)	6,323.83 ± 156.06	5,336.22 ± 316.13	0.003*
Maximum extension position (*V*_*1b*_, *V*_*2b*_)	9,186.58 ± 512.61	6,735.46 ± 794.63	0.003*

Airway volume increased in the upper airway filling model as the body position varied from the supine to maximum extension position (Group A: volume increase 2,953.75 ± 524.6 mm^3^, rate of change 31%; Group B: volume increase 1,632.89 ± 662.66 mm^3^, rate of change 25%; *p* = 0.052). Group A had a higher rate of volume change compared to Group B (Table 3). However, the difference was not statistically significant.

**Table 3:** Volume difference in the upper airway filling model between the two groups in the supine and maximum extension positions

Group	Item	Difference (mm^3^)	Rate of change (%)	*p*-value
Group A	*V*_*1b*_ − *V*_*1a*_	2,953.75 ± 524.6	31	0.052^#^
Group B	*V*_*2b*_ − *V*_*2a*_	1,632.89 ± 662.66	25	0.052^#^

### Comparison of morphological data on three planes

In the coronal plane, there was no significant between-group difference (*p* = 0.091) in the rate of change (less than 3%) of the longitudinal radial distance (Table 4). The projection areas increased by approximately 16% with variation in body positions in both groups (Table 4). The change rates of the lower corner angles in both groups were approximately 10%; the left corner angle change rate was 25% (Table 4).

**Table 4:** Morphological measurements in the coronal plane projections of a 3D model of gas filling

Item	Group	Supine position	Maximum extension position	Rate of change (%)	Difference	*p*-value
**Coronal plane projections**						
*L*_*STC*_/*L*_*ETC*_* (mm)*	A	44. 10 ± 2. 34	47.15 ± 1.54	6. 00	3.25 ± 1.28	0.078
	B	38. 33 ± 3.10	40.15 ± 2.91	7. 00	3.18 ± 0.98	
*L*_*SLC*_/*L*_*ELC*_* (mm)*	A	7.99 ± 2. 15	8.13 ± 1.85	3. 00	1.17 ± 0. 87	0.091
	B	8.01 ± 1.15	8.11 ± 2.10	1. 00	1.03 ± 0. 91	
*S*_*SH*_/ *S*_*EH*_* (mm*^*2*^*)*	A	362. 60 ± 30.15	438.34 ± 56.85	17.00	68. 39 ± 15.87	0.058
	B	312. 01 ± 45.35	373.28 ± 39.24	16.00	64.19 ± 18.61	
*α *_*SIC*_/*α *_*EIC*_	A	88.33 ± 17. 34°	98.11 ± 18. 55	10.00	9.25 ± 1.28°	0.067
	B	79.49 + 16. 73°	87. 65 ± 18.91°	9. 00	8.57 ± 0. 98°	
*α *_*SLC*_/*α *_*ELC*_	A	73.35 ± 19. 23°	71. 74 ± 20. 34°	25.00	2.01 ± 0.15°	0.113
	B	68.21 ± 18. 33°	66. 37 ± 18.01°	26.00	1.99 ± 0.27°	
**Sagittal plane projections**						
*L*_*STS*_/*L*_*ETS*_* (mm)*	A	38.12 ± 2.34	65.95 ± 1.54	42.00	29.2 ± 1.28	0.003
	B	35.31 ± 3.10	37.28 ± 2.91	5.00	3.18 ± 0.98	
*L*_*SLS*_/*L*_*ELS*_* (mm)*	A	8.96 ± 2.34	15.94 ± 2.54	30.00	6.25 ± 1.28	0.040
	B	3.31 ± 3.10	11.28 ± 2.91	25.00	7.18 ± 0.90	
*S*_*SH*_/ *S*_*EH*_* (mm*^*2*^*)*	A	218.60 ± 30.15	324.3 ± 56.85	33.00	100.3 ± 37.083	0.003
	B	199.01 ± 45.35	223.2 ± 39.24	6.00	25.19 ± 6.61	
*α *_*SIS*_/*α *_*EIS*_	A	127.33 ± 27.34°	168.3 ± 33.51°	24.00	41.25 ± 8.28°	0.027
	B	132.82 ± 26.73°	165.6 ± 28.91°	14.00	30.57 ± 7.98°	
*α *_*SLS*_/*α *_*ELS*_	A	18.12 ± 4.31°	35.32 ± 11.61°	48.00	18.14 ± 3.56°	0.198
	B	16.16 ± 3.11°	31.64 ± 9.27°	48.00	16.79 ± 0.16°	
**Horizontal plane projections**						
*L*_*STH*_/*L*_*ETH*_* (mm)*	A	44.10 ± 6.34	26.45 ± 1.54	40.00	18.25 ± 1.28	0.075
	B	38.33 ± 3.10	23.15 ± 2.91	39.00	16.28 ± 0.98	
*L*_*SLS*_/*L*_*ELS*_* (mm)*	A	24.59 ± 2.15	22.53 ± 1.85	8.00	1.17 ± 0.87	0.121
	B	21.67 ± 1.15	19.89 ± 2.10	8.00	1.03 ± 0.91	
*S*_*SH*_/ *S*_*EH*_* (mm*^*2*^*)*	A	837.60 ± 45.15	700.34 ± 46.85	16.00	140.39 ± 17.87	0. 061
	B	727.51 ± 45.35	583.28 ± 40.24	18.00	138.19 ± 18.61	
*α *_*SIH*_/*α *_*EIH*_	A	81.33 ± 17.34°	90.11 ± 18.55°	10.00	9.25 ± 1.28°	0.093
	B	79.49 ± 16.73°	87.65 ± 18.91°	9.00	8.57 ± 0.98°	
*α *_*SLH*_/*α *_*ELH*_	A	102.71 ± 14.81°	108.45 ± 15.37°	5.00	7.01 ± 1.13°	0.211
	B	100.56 ± 15.36°	105.79 ± 16.33°	5.00	6.73 ± 1.45°	

In the sagittal plane, a 42% increase in length was detected in Group A, while a 5% increase was detected in Group B (Table 4). The longitudinal radial length increased by 30% and 25% in Groups A and B, respectively (Table 4). A 33% increase of the projection area was detected in Group A, while a 6% increase was detected in Group B (Table 4). The left corner angle of the projection quadrilateral increased to 48% in both groups with variation in body position. (Table 4).

In the horizontal plane, the increase of transversal radial length approached 40% and 39% in Groups A and B, respectively, while there was an 8% increase in longitudinal radial length (Table 4). The projection area in this plane increased by 18% as the body position changed (Table 4).

## Discussion

Airway assessment can be used to quantify the morphological features of an upper airway with complicated geometry, which is a key step prior to performing general anesthesia. Complications associated with improper airway management are the most common cause of morbidity and mortality [16]. As such, difficulties in establishing a clinical airway are an ongoing challenge for anesthesiologists [2]. Clinical practice guidelines recommend specific strategies to ensure patient safety during the management of difficult or unexpected airway. However, no gold standard method has been published [17]. Visibility-enhancing applications, such as fiberoptic bronchoscopy and video laryngoscopy, have greatly reduced the incidence of intubation challenges of difficult airway during the perioperative period. However, none of these techniques can fully eliminate the risk. Image-based 3D models developed in this study display the anatomical structure in detail, and thus provide a novel cutting-edge digital technology to detect potential risks of difficult airway.

Rapidly developing medical imaging techniques, such as ultrasound, computed tomography (CT), and MRI, that generate images of internal organs and tissues have significantly improved clinical diagnoses. Kelly et al., [18] created a resin model of the nasal cavity based on computerized x-ray images to study the distribution of the gas-flow field within the nasal cavity. Chun et al., [19] developed an MRI-based kinetic model for the upper airway of rats to investigate muscle characteristics, airway shape, and anatomical structure to be applied to patients with obstructive sleep apnea (OSA). Yu et al., [20] created a 3D finite element model of airway filling based on spiral CT images of healthy individuals and patients with obstructive sleep apnea–hypopnea syndrome (OSAHS) using a surface rendering method. Using this model, the original shape of the upper airway was accurately preserved, and the airflow of the entire respiratory cavity was numerically simulated using finite element analysis. Zhou et al., [21]reconstructed a 3D model of the upper airway of patients with small-jaw deformity and OSAHS and further measured the minimum cross-sectional area of the upper airway-related sagittal, cross-sectional, and coronal planes. Upper airway stenosis of patients with OSAHS was mainly detected in the sagittal plane, with the most marked stenosis in the pharyngeal segment. Liu et al., [22]constructed an upper airway 3D finite element model based on spiral CT data from 10 patients with OSA and performed fluid dynamic simulation to assist clinical diagnosis and treatment.

However, despite these advances, there are currently only a few published studies addressing finite element analysis of the upper airway. Fan et al., [23] used 3D CT to observe changes in the intrinsic oral volume based on the tongue position in patients with a difficult airway and found an increased volume ratio between the front of the tongue before and after sticking out the tongue. Most of the current research is based on CT scans, which are insufficient for identifying airway soft tissue and other structures. Therefore, in this study we used MRI imaging to create an upper airway filling 3D model in which soft tissues can be clearly identified.

The morphological data measured in the developed model agree with published studies [17]. The model precisely depicted detailed morphological structure and further investigated the anatomical specificity of the difficult airway group. Volumetric values of the upper airway changed with variation in body positions. Volume increased in both normal and difficult airway as patients moved from the supine to maximum elevation position. Difficult airway showed a relatively low increase in volume rate. These findings agree with a published study addressing different intubation oropharyngeal models [24].

The special anatomical features of the upper airway technically support our results. The muscular structures in the oropharynx (e.g., soft palate, tongue, PA) play a key role in supporting the upper airway due to the absence of bony structures. Therefore, the airway opening is controlled by not only bony structures, such as the mandible and teeth, but also relevant soft tissues. Anatomical positions of those tissues are movable with variations in body position. The increased volume of the upper airway favored the placement of the laryngoscope to expose the glottis and smooth insertion of the endotracheal tube during endotracheal intubation.

Morphological data of the sagittal plane play a key role in assessing the potential occurrence of a difficult airway. Morphological datasets on three projection planes proposed in this study quantified the irregular geometry of an upper airway. Technically, four sets of geometric data—maximum longitudinal radial distance, maximum transverse radial distance, surface area, and corner angles—were associated with volumetric changes. Those data showed marked changes on the sagittal planes with respect to variation in body position, while no obvious changes were present in the coronal and horizontal planes. It should be noted that an upper airway filling 3D model can display an irregular geometric shape. We simplified the geometric expression for the 3D model to four sets of data in our analysis. Similar methods have been used in a knee meniscus model [14, 15]. The above-mentioned results demonstrated that the morphological variation of the upper airway in the sagittal plane was an appropriate indicator of a difficult airway when a patient moves from the supine position to maximum elevation. Image-based morphological assessments can initially screen patients with a high risk of a difficult airway and can further provide comprehensive evaluation using indicators such as the Mallampati score, Cormack-Lehane classification, and Wilson’s score, as well as physical features like mouth opening less than 30 mm, TMD less than 60 mm, and restrictions of neck flexion and extension. 

Several limitations of this study should be mentioned. First, the technique in our study indicated free volume in the airway, but this volume also varied when the soft tissue was "crushed" by the intubation tool. Secondly, statistical significance of dimensional changes may be difficult to correlate with clinical correlates of intubation or ventilation difficulties because there is no reliable and accurate method to evaluate and predict difficult airway at present. Thirdly, this study only included a single group of patients, and thus these results may not apply to patients with trauma, head and neck pathologies, or prior difficulty. Moreover, this was a retrospective study with a small patient sample size. Large sample multi-center and randomized controlled trials should will be conducted in future studies to better inform actual clinical application. Finally, the use of this digital assessment technique in daily practice is limited, which was mainly used to evaluate the impact of position-specific morphological changes in difficult airway difficult airway when difficult intubation is highly suspected or in cases of compressive tumor airway surgery.

## Conclusion

The results of this study demonstrate that the 3D finite element model of upper airway filling based on MRI 2D image reconstruction can effectively reflect the anatomy of the upper airway. The airway length, area, and angle changes of the longest diameter line in the sagittal position can reflect the anatomical specificity of patients with difficult airway. Therefore, the image-based modeling technique developed in this study can be used to quantify the morphological features of an upper airway with complicated geometry. The proposed morphological dataset defining multi-view projections simplified the key geometric features of an upper airway and a difficult airway. Morphological modifications in the sagittal plane revealed the anatomical specificity of a difficult airway as the patient moved from the supine to the maximum extension position. These findings provide new guidance for the evaluation and prediction of difficult airway during clinical anesthesia.

## Supplementary Information

**Additional file 1.**

## Data Availability

The data supporting the findings of this study are not publicly available because of institutional policy but are available from the corresponding author upon reasonable request.

## Abbreviations

3D: Three-dimensional
CT: Computed tomography
MRI: Magnetic resonance imaging
OSA: Obstructive sleep apnea
OSAHS: Obstructive sleep apnea–hypopnea syndrome
2D: Two-dimension
TMD: Thyromental distance
