# Supplementary material for: Morphological specificity analysis of an image-based 3D model of airway filling in a difficult airway
Source: BMC Anesthesiol. 2022 Nov 3;22:336. doi: 10.1186/s12871-022-01880-6 (PMC9632020; doi:10.1186/s12871-022-01880-6)
Supplement: Supplementary file 1 — Additional file 1. [file 12871_2022_1880_MOESM1_ESM.doc]

**Supplementary figure 1.** A. Supine position; B. Maximum extension position. OA: Oral axis; PA: Pharyngeal axis: LA: Laryngomatic axis


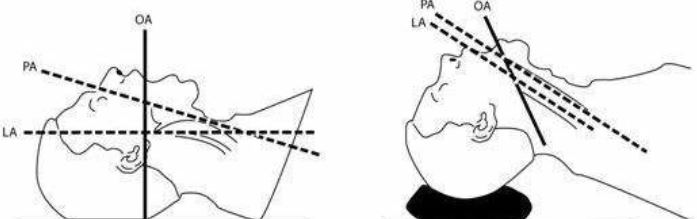


**A B**

Supplementary table 1. Participant information

| Groups | ASA  classification  (number, Ⅱ/Ⅲ) | Age  (years, x±s) | BMI  (kg/m2, x±s) | Gender  (number, Men/  Women) |
| --- | --- | --- | --- | --- |
| Normal airway (Group A) | 18/2 | 39±3.6 | 21.3±2.5 | 14/6 |
| Difficult airway (Group B) | 19/1 | 41±2.9 | 23.8±6.2 | 17/3 |

General data of the two groups (n=20)
